# Role of Choline in Ocular Diseases

**DOI:** 10.3390/ijms22094733

**Published:** 2021-04-29

**Authors:** Jin-Sun Hwang, Young-Joo Shin

**Affiliations:** Department of Ophthalmology, Hallym University Medical Center, Hallym University College of Medicine, Seoul 07442, Korea; hotsayme@naver.com

**Keywords:** choline, ocular disease, dry eye syndrome, retina, diabetic retinopathy, glaucoma

## Abstract

Choline is essential for maintaining the structure and function of cells in humans. Choline plays an important role in eye health and disease. It is a precursor of acetylcholine, a neurotransmitter of the parasympathetic nervous system, and it is involved in the production and secretion of tears by the lacrimal glands. It also contributes to the stability of the cells and tears on the ocular surface and is involved in retinal development and differentiation. Choline deficiency is associated with retinal hemorrhage, glaucoma, and dry eye syndrome. Choline supplementation may be effective for treating these diseases.

## 1. Introduction

Choline is important for maintaining the structure and normal function of cells [1]. It is a precursor of acetylcholine, phosphatidylcholine, and methyl-donor betaine [1,2]. The metabolites of choline include trimethylamine N-oxide, betaine, choline, phosphocholine, glycerophosphocholine, phosphatidylcholine, sphingomyelin, lysophosphatidylcholine, and acetylcholine (Ach) [3]. Choline is involved in retinal development and differentiation. Choline deficiency has been reported to be associated with retinal diseases, glaucoma, dry eye syndrome, and disorders of the lens, optic nerve, and the visual cortex of the brain. Choline supplementation may protect the eye from diseases and be an effective treatment for various eye diseases.

## 2. Choline in Dry Eye Syndrome and Ocular Surface

Dry eye syndrome is the most prevalent ocular disease globally [4]. It is characterized by eye discomfort, functional visual disturbances, tear film instability, and ocular surface alterations [5,6]. The tear film consists of three layers: the lipid layer on the outside, the aqueous layer in the middle, and the inner mucus layer [7]. Dry eye syndrome can be classified into the aqueous-deficient and evaporative etiological subtypes [8]. Aqueous deficient dry eye syndrome results from the reduction in aqueous tear production, and evaporative dry eye syndrome is characterized by excessive evaporation of the tear film resulting from the lack of a protective lipid layer [8]. The lacrimal functional unit, which includes the cornea, conjunctiva, meibomian glands, and the main and accessory lacrimal glands, is useful in understanding dry eye and tear dynamics [9]. Lacrimal glands, which secrete electrolytes, water, proteins, and mucins known as lacrimal gland fluid into the tear film, are innervated by parasympathetic and sympathetic nerves [10]. The parasympathetic system predominantly regulates the secretions of lacrimal glands [10]. The parasympathetic nervous system releases ACh, which acts on muscarinic and nicotinic receptors [11]. Muscarinic receptors are important mediators of secretions by the lacrimal and salivary glands [12]. Muscarinic receptors have also been reported to be present in the cornea, conjunctiva [13], and meibomian glands [14], suggesting that muscarinic receptors may affect secretions by these tissues. Choline is a precursor of Ach, which is a neurotransmitter of the parasympathetic nervous system [15]. Choline deficiency may contribute to the pathophysiology of dry eye syndrome by the reducing parasympathetic tone. 

Another important pathological mechanism of dry eye syndrome is inflammation [16]. Conjunctival inflammation manifests as T-cell infiltrates and the upregulation of cluster of differentiation (CD) 3, CD4, and CD8, as well as lymphocyte activation markers such as CD11a and human leukocyte antigen (HLA)-DR. Pro-inflammatory cytokines, such as interleukin (IL)-1, IL-6, IL-8, and tumor necrosis factor-α, have been reported to be elevated in the ocular surface [17,18]. Choline deficiency has been reported to cause oxidative damage in various organs [19]. Thus, choline deficiency may enhance inflammation in dry eye syndrome and delay wound healing on the ocular surface. Choline supplementation suppresses tissue inflammation and oxidative damage [20]. Choline supplementation has been suggested for use as a new therapeutic method for controlling the immune inflammation [21]. Nicotine receptors stimulated with ACh in inflammatory cells inhibit the release of pro-inflammatory cytokines in a concentration-dependent manner [22]. It has also been reported that choline is involved in cell damage and metabolism, protects against hypoxia-induced injuries of vessels and vascular endothelial cells [23], and is essential for tissue repair after injury [24]. 

The ocular surface is a gateway exposed to the external environment, and it receives external stimulation [25]. Various nutrients and immune-related components are supplied to the surface of the eyeball through the tear film, and various immune systems including neutrophils and lymphocytes, are active [26]. There are two types of immune systems: innate and adaptive [27]. Innate immunity fights infection with high efficiency during the early stages after an encounter with external stimuli until other immune mechanisms work [28]. Innate immunity involves epithelial cells, fibroblasts, natural killer cells, macrophages, neutrophils, dendritic cells, mast cells, basophils, eosinophils, mucin, and lysozyme [29]. Corneal and conjunctival epithelial cells secrete the inflammatory cytokines, including tumor necrosis factor-α, IL-1, IL-6, and IL-8, in response to immune stimuli. Adaptive immunity is antigen-dependent, and it includes T- and B-lymphocytes [30]. Innate immunity is required to eliminate harmful pathogens and may contribute to tissue remodeling after injury; however, if not properly regulated, this type of immune response can cause persistent inflammation, which can impair organ function [28]. Neutrophils remove external pathogens by forming neutrophil extracellular traps (NET) for large external pathogens in the cell nucleus and mitochondria (NETosis), which are also involved in autoimmune and ocular surface inflammatory diseases. In addition to building resistance to external pathogens, neutrophils on the ocular surface are also involved in various inflammatory reactions. In recent years, it has been found to affect dry eye syndrome and autoimmune diseases. It has been reported that choline tends to reduce plasma haptoglobin concentrations, suggesting that choline reduces systemic inflammation [31]. Choline modulates the function of inflammatory cells and elevates the mRNA levels of enzymes and receptors involved in metabolism [32]. Impaired choline uptake or phosphorylation reduces mitochondrial adenosine triphosphate (ATP) synthesis, which leads to AMP-activated protein kinase (AMPK) activation and induces mitophagy causing inflammation [33].

Ocular pain and discomfort are the characteristic symptoms of dry eye syndrome. The main symptoms of dry eye syndrome include dryness, itchiness, burning, stinging, grittiness, foreign body sensation, tearing, tired eyes, redness, and blurred vision [7]. Pain is a result of the network activity of multiple areas related to sensory, cognitive, and emotional functions [34]. Pain pathways in the central nervous system are modulated by the cholinergic system [35]. There are two types of ACh: muscarinic and nicotinic receptors. Muscarinic acetylcholine receptors are ubiquitously found in the cerebral cortex [35]. The perception of pain is modified by cholinergic transmission [35]. Choline has an analgesic effect by activating α7 nicotinic receptors [36]. The antinociceptive effects of cholinergic agonists are related to gamma-aminobutyric acid-ergic (GABAergic) signaling, as well as nicotinic and muscarinic modulation of nociceptive transmission [37]. Choline deficiency may contribute to the development of ocular symptoms associated with dry eye syndrome. 

**Figure 1:**
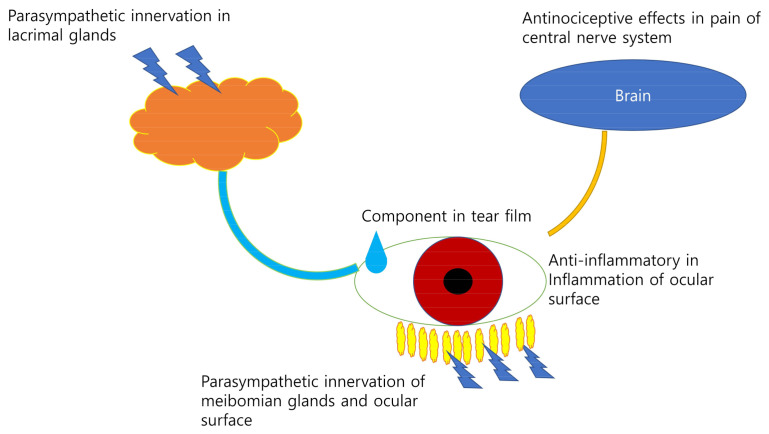
The role of choline in dry eye syndrome. Choline may affect dry eye syndrome by parasympathetic innervation of the meibomian glands and the ocular surface, a component in the tear film, exerts antinoiceptive effects in pain on central nervous system and anti-inflammatory in inflammation of ocular surface.

Choline is a precursor of phosphatidylcholine and a component of the tear film [7]. Choline alfoscerate is a surfactant that can stabilize the aqueous and lipid layers of the tear film. The effect of oral choline alfoscerate has been reported in patients with dry eye syndrome [38]. Choline alfoscerate may affect dry eye syndrome mainly through antinociception in the central nervous system and an improvement in tear stability as an emulsifier in tears [38]. Choline alfoscerate, in combination with a cholinesterase inhibitor, may have a sufficient effect on tear secretion [39]. A schematic diagram of this process is shown in Figure 1.

Betaine, which is an intermediate of choline, has been reported to reduce corneal staining and protect the ocular surface integrity against the environmentally dessicating stress as an osmoprotectant and an anti-inflammatory agent [40,41]. Betaine decreases tear osmolarity and inflammatory cytokines, including tumor necrosis factor-α, IL-1, IL-8, C-C motif chemokine ligand 2 (CCL2), and C-C motif chemokine ligand 20 (CCL20) [40,41]. Tear hyperosmolarity is an essential mechanism underlying dry eye syndrome as a potent inflammatory stress that impairs normal cell functions of corneal and conjunctival epithelial cells [40,41].

Meibomian glands, which are located in the eyelids, secrete lipids or meibum unto the ocular surface [42]. Hyperkeartinization and obstruction of the meibomian gland ducts result in meibomian gland dysfunction with meibocyte atrophy and peroxisome proliferator-activated receptor gamma alteration [43]. Meibomian gland dysfunction is the most common cause of the evaporative dry eye syndrome [42]. The meibum includes wax, diglycerides, triglycerides, hydrocarbons, free fatty acids, sphingomyelin, phosphatidylcholine, and ceramides [44]. Phospholipids comprise only a small portion but, as surfactants, the lipid ingredients are well distributed over the tear film [43]. Sphingomyelin is metabolized sequentially into ceramide, sphingosine, and sphingosine-1-phosphate [45]. The quality of meibum is related to the changes in its sphingolipids in meibum [46]. Ceramide and sphingosine induce cell cycle arrest, senescence, apoptosis, and cell differentiation [47]. Ceramides and triglycerides have been reported to be elevated in dry eye syndrome in response to tear hyperosmolarity and to facilitate the secretion of inflammatory cytokines [48]. Neutral sphingomyelinase 2 may play a pivotal role in cellular responses to hyperosmolar stress, including cytokine secretion and lipid droplet formation following the production of ceramide [48]. Sphingosine-1-phosphate promotes cell survival, proliferation, migration, anti-apoptosis, and angiogenesis [49]. Sphingosine-1-phosphate modulates inflammatory responses, targets histone deacetylase and human telomerase reverse transcriptase, and triggers canonical G protein-coupled receptor signaling such as Rac, extracellular signal-regulated kinases, phosphatidylinositol-3 kinase/Akt, phospholipase C, and Rho pathways by binding to sphingosine-1-phosphate receptors [50,51,52]. Fingolimod (FTY720), a sphingosine-1-phosphate receptor inhibitor, has been shown to improve the dry eye syndrome by inhibiting leukocyte migration, the extracellular signal-regulated kinase signaling pathway, and inflammatory cytokine secretion [53]. 

Choline acetyltransferase and acetylcholinesterase have been found in the corneal epithelium [54]. Cholinergic signaling stimulation of the corneal epithelium promotes the directional migration of corneal epithelial cells and wound healing through muscarinic acetylcholine receptors (mAChRs) and nicotinic acetylcholine receptors (nAChRs) [55]. Choline acetyltransferase is completely regenerated 28 days after corneal abrasion [56]. Citicoline eye drops were applied to the recovery of corneal sensitivity after laser in situ keratomileusis by enhancing corneal innervation [57]. Choline salicylate has been developed to increase the viscosity of eye drops [58]. Nitrogen mustard, which is a vesicant responsible for irreversible corneal injury, damages the corneal epithelium and stroma through the sphingomyelin–ceramide pathway [59]. Detergent chemicals, widely used in household products, are toxic, and they induce the inflammation via the alteration of phospholipid and sphingolipid metabolism [60]. The mechanisms of choline in dry eye syndrome are summarized in Table 1.

## 3. Choline in the Retina

The retina is composed of multiple layers of cells that receive light and convert them to signals that are transferred to the brain [61]. There are five types of cells in the retina: photoreceptors, bipolar cells, ganglion cells, horizontal cells, and amacrine cells [62]. Retinal diseases can result in blurred vision and blindness [63]. The development of a treatment modality for retinal disease is important for preserving vision and protecting against blindness. 

Choline is an essential nutrient that is necessary for optimal brain development [64]. The retina is derived from the neuroepithelium of the ventral diencephalon [65]. Choline also facilitates optimal retinal development [66,67]. Choline acetyltransferase-expressing retinal amacrine cells exist in the retinas of human and vertebral animals [68]. During retinal development, choline acetyltransferase is expressed in both the inner and outer plexiform layers during embryonic life in the zebrafish, and choline acetyltransferase-stained amacrine cells are found only after hatching [69]. The choline acetyltransferase in the outer plexiform layer of the retina slowly reduces during larval development [69]. Choline plays a critical role in the regulation of the temporal progression of retinogenesis, and adequate choline supplementation is essential for optimal development of the visual system [67]. Choline deficiency leads to impairments in the differentiation of retinal neuronal cells, such as the densities of early born retinal ganglion cells, amacrine and horizontal cells, and cone photoreceptor precursors [67]. Furthermore, choline deficiency during retinogenesis causes the persistent retinal cytoarchitectural defects, ranging from focal lesions with the displacement of retinal neurons into the subretinal space to severe hypocellularity and ultrastructural defects in photoreceptor organization [67]. 

ACh is a major retinal neurotransmitter that modulates visual processing through a lot of functions of cholinergic receptors found in various retinal cells [70]. ACh is secreted from retinal amacrine cells under scotopic visual conditions [70]. Choline acetyltransferase is expressed in retinal amacrine cells and retinofugal fibers of the optic nerve across vertebrates [68,71]. Acetylcholinesterase (AChE) is found in the inner plexiform layer of the retina [72]. AChE is observed at the synaptic and non-synaptic sites. The reaction product from AChE is found in amacrine cells, bipolar cells, ganglion cells, and Müller cells [73]. Distinct distributions of choline acetyltransferase (+) vs. AchE (+) cells in the inner half of the retina provide graded distributions of ACh, which can direct cell differentiation and network formation [74]. The cholinergic differentiation of neonatal rat retinal cells in vitro is stimulated by IL-4 [75]. Acetylcholine-related molecules in the retina are implicated in the communication between photoreceptors and the retinal pigment epithelium [76].

Choline is used exclusively in mammalian photoreceptor cells for phospholipid synthesis [77]. Citicoline, which is known as cytidine diphosphate-choline (CDP-Choline) or cytidine 5’-diphosphocholine and is an intermediate in the synthesis of phosphatidylcholine [78], has a protective effect on damaged retinal ganglion cells in the mouse culture retina [79]. It has been reported as a cytoprotective molecule in age-related macular degeneration [80]. Citicoline has been suggested as a possible agent for neuroprotective and regenerative therapies [78]. 

Age-related macular degeneration is a degenerative disease characterized by photoreceptor apoptosis, retinal pigment epithelium atrophy, and pathological neovascularization [81,82]. Ceramide is an essential second messenger for the activation of apoptosis in photoreceptors [83]. The inhibition of ceramide production protects retinal cells from apoptosis [84]. Sphingosine-1-phosphate may be involved in the pathogenetic mechanisms underlying age-related macular degeneration, such as fibrosis, inflammation, neovascularization, and Müller glial cell migration through the trans-activation and production of vascular endothelial growth factor, fibroblast growth factor, platelet-derived growth factor, and other growth factors [85]. In contrast, sphingosine-1-phosphate promotes the survival of photoreceptors and ganglion cells [82]. Betaine is associated with a reduced risk of age-related macular degeneration and may protect the retina [86]. 

## 4. Choline in Retinal Vessels

Retinal vessels are impermeable and form a blood–retina barrier [87]. Vascular endothelial cells of the retina adhere to each other tightly and have tight junctions, which prevents the leakage of plasma proteins from the vessels [88]. Blood–retina barrier breakdown leads to macular edema, which occurs in various retinal diseases and is linked to visual disturbances [89]. Choline and its metabolites have a protective effect on various stress-induced injuries of vessels and vascular endothelial cells [23].

Choline deficiency induces ocular hemorrhagic lesions after the development of renal necrosis [90]. Choline effectively attenuates brain ischemic injury through ACh-mediated vascular endothelium-dependent vasodilatation [91]. Cardiovascular damage is attenuated by choline through the enhancement of vagal tone, which results in heart rate reduction, vasodilation of vessels, and glandular activity in the heart, lungs, and digestive tract, among others, and suppresses the inflammatory response in hypertensive rats [92]. This suggests that choline has cardiovascular protective effects and can be used as adjuvant therapy for hypertension [92]. However, higher plasma phosphatidylcholine concentrations are associated with both a favorable cardiometabolic risk factor profile, including higher concentrations of high-density lipoprotein (HDL) cholesterol, lower body mass index (BMI), lower odds of hypertension and diabetes, and an unfavorable profile including higher concentrations of low-density lipoprotein (LDL) cholesterol and triglycerides [93]. Higher plasma betaine levels are associated with a favorable profile, such as lower LDL cholesterol and lower odds of diabetes [93]. 

Diabetic retinopathy is a severe microvascular disease that is a complication of diabetes mellitus [94] and is the primary cause of visual loss through pathologic neovascularization [95]. Diminished retinal blood supply, an early sign of diabetic retinopathy, is associated with deficits in both neuronal and vascular elements [96]. Deficits in cholinergic cells are linked to reduced blood flow in diabetic retinopathy [97,98]. ACh is a potent vasodilator secreted by cholinergic amacrine cells. The vascular targets of ACh are muscarinic type 3 receptors (m3AChRs) expressed on both vascular endothelial cells and pericytes [99]. The optogenetic stimulation of cholinergic amacrine cells enhances retinal capillary blood supply in diabetic retinopathy [98]. Betaine inhibits pathologic vascularization via the suppression of Akt in the retinas of streptozotocin-induced hyperglycemic rats, which suggests that betaine can be useful as a potential therapeutic approach for delaying the onset of diabetic retinopathy complications by inhibiting pathologic retinal neovascularization in patients with diabetes [100]. Betaine is obtained through diet or the oxidation of choline, which is its precursor [101]. Betaine, an intermediate of choline synthesis, has an anti-angiogenic effect on retinal neovascularization in oxygen-induced retinopathy by reducing reactive oxygen species, vascular endothelial growth factor, and Akt signaling [102]. Citicoline (cytidine 5-diphosphocoline), which is an intermediate in the synthesis of phosphatidylcholine [78], has anti-apoptotic effects, increases retinal dopamine concentrations, and counteracts retinal nerve fiber layer thinning [103]. However, topical citicoline drops had no significant effect on the superficial and deep microvascular structures of the retina or choriocapillaris [104].

Ocular ischemic syndrome is a rare severe ocular disease caused by ocular hypoperfusion due to stenosis or occlusion of the common or internal carotid artery [105]. In patients with ocular ischemic syndrome, a more rapid and stable improvement in visual acuity was observed in the choline alfoscerate group [106]. 

Sphingosine-1-phosphate plays an essential role in angiogenesis through Rho and Rac GTPases [107,108]. The inhibition of sphingosine-1-phosphate production reduces vascular endothelial growth factor-induced retinal endothelial cell proliferation and retinal vascular leakage [109]. Acid sphingomyelinase activation is involved in diabetic retinopathy [110]. Mitochondrial ceramide levels are elevated in diabetic retinopathy, which is associated with higher acid sphingomyelinase levels [110]. Blocking acid sphingomyelinase protects mitochondrial function by regulating the number of mitochondria and the formation of reactive oxygen species [110]. The mechanisms of choline in retina and retinal vessel diseases are summarized in Table 2. 

## 5. Choline in the Optic Nerve

The optic nerve connects the eye to the brain and transfers visual information from the retina to the visual cortex of the brain [111]. Representative diseases of the optic nerve are glaucoma and optic neuropathy, which are characterized by visual disturbances and specific visual field defects [111].

Glaucoma is a neurodegenerative disease that affects primary optic neuropathy, with secondary effects in the central nervous system [112]. The pathophysiological mechanisms of glaucoma include high intraocular pressure, a disruption in ocular blood flow, systemic hypotension, obstructive sleep apnea/hypopnea syndrome, and neurovascular dysregulation [113]. Glaucoma has been classified as open-angle glaucoma and angle-closure glaucoma [114]. High-tension glaucoma and angle-closure glaucoma are characterized by high intraocular pressure and changes in the optic nerve due to ocular pressure [114]. Normal-tension glaucoma may be suggested to be related to the autoimmune, genetic, or systemic pathogenesis of the optic nerve because the intraocular pressure is normal [113]. The cholinergic nervous system plays an important role in neurocognitive functions in the brain and visual neurophysiology [115]. ACh is crucial for the survival and viability of retinal ganglion cells [115]. Cholinergic medications have been used for the treatment of glaucoma. Cholinergic agonists such as ACh, pilocarpine, carbacol, and echothiophate can lower intraocular pressure by pupil constriction, or miosis, which reduces the resistance to aqueous humor outflow directly or indirectly [115]. Citicoline, which is an intermediate in the synthesis of phosphatidylcholine [78], protects against the progression of visual field defects in glaucoma patients [116,117]. Citicoline treatment increases retinal dopamine concentration in rabbits [118] and rescues retinal ganglion cells following partial optic nerve crush in rats [119]. It restores mitochondrial functions, promotes the synthesis of Ach and myelin, stimulates antioxidant activity, and prevents neuronal cell death [115]. Topical citicoline has been suggested to have neuroprotective action, although it does not affect retinal vasculature and the choriocapillaris [103,117].

Non-arteritic ischemic optic neuropathy, which is an irreversible, painless, and acute ischemic disease of the optic nerve, is characterized by sudden vision loss and visual field loss [120]. Citicoline treatment promotes neuroenhancement by improving retinal ganglion cell function and neural conduction along visual pathways and demonstrates neuroprotection in human models of non-arteritic ischemic optic neuropathy involving fast retinal ganglion cell degeneration by unmodified or improved retinal nerve fiber layer conditions [121].

Cholinergic neurons, which are defined as choline acetyltransferases, are localized in the cerebral cortex [122]. Choline acetyltransferase (+) cells are distributed throughout cortical layers II-VI, although they are mostly concentrated in layers II-III [122]. Choline acetyltransferase (+) cells, with abundant branches and varicosities, are localized in all layers of the visual cortex, suggesting that they may have functional contributions to the visual cortex [123]. Cholinergic fibers may mediate cortical processing by controlling synaptic transmission and plasticity [124]. Cholinergic enhancement promotes function and plasticity in the visual cortex [125] and attenuates perceptual suppression during binocular rivalry; therefore, it reduces the overall rate of interocular competition while enhancing the visibility of superimposition mixed percepts [126]. Cholinergic neurons have a strong potential to enhance visual perception [127]. The mechanisms of in visual pathway are summarized in Table 3.

## 6. Choline in the Lens

The lens contains lipids such as phosphatidylethanolamine, phosphatidylcholine, dihydrosphingomyelin, and phosphatidic acid [128,129]. Lens lipids contribute to maintaining lens transparency, and changes in lens lipid composition with aging contribute to cataracts [128]. Lipid oxidation has been suggested to lead to a significant loss of unsaturated phospholipids, including phosphatidylcholine, and the relative abundance of dihydrosphingomyelin with age and during cataract [129,130]. Presbyopia, which results from lens hardening, is characterized by loss of accommodation, and it is managed by the wearing of near or reading glasses. Topical lipoic acid choline ester eye drops have been reported to improve near visual acuity in the patients with presbyopia in a mouse study and a preliminary efficacy trial [131,132]. Cataract, a lens opacity, is characterized by progressive visual loss and the requirement for surgical intervention [133]. The α-crystallin of the lens interacts with phospholipids and phosphocholine [134]. A decrease in phosphocholine synthesis is linked to swelling and opacification of the lens in rats [133]. 

## 7. Conclusions

Choline is involved in the development and differentiation of the retina and the eye. Choline and its metabolites perform various functions in the visual pathway, which includes the ocular surface, retina, optic nerve, and visual cortex of the brain. Choline deficiency is associated with retinal hemorrhage, glaucoma, and dry eye syndrome. Choline and its metabolite supplementation may be effective in treating these diseases. 

## Figures and Tables

**Table 1:** Role of choline and choline metabolite in dry eye syndrome.

References	Mode of Action or Mechanism	Organ
Dartt 2009 [10]	Parasympathetic innervation	Lacrimal glands
Naser et al., 2018, Kusuda et al., 2020 [35,37]	Pain signaling pathway	Cholinergic nervous system in brain
Zhou et al., 2012, Choi et al., 2020 [7,38]	Components of tear film	Ocular surface
Chen et al., 2013, Hua et al., 2015, Robciuc et al., 2012 [40,41,48]	Tear osmolarity and inflammation	Ocular surface
McCulley et al., 2003, Paranjpe et al., 2019 [44,46]	Components of meibum	Meibomian glands
Cinar et al., 2019 [57]	Wound healing	Ocular surface

**Table 2:** Role of choline and choline metabolite in retina and retinal vessel diseases.

References	Mode of Action or Mechanism	Disease
Trujillo-Gonzalez et al., 2019 [67]	Differentiation during retinogenesis	Retinal cytoarchitectural defects
Bikbova et al., 2017 [78]	Neuroprotection	Age-related macular degeneration
German et al., 2006 [84]	Anti-apoptosis	Age-related macular degeneration
Spiegel et al., 2003 [85]	Protection against inflammation, fibrosis and neovascularization	Age-related macular degeneration
Nakazawa et al., 2007; Ivanova et al., 2020 [97,98]	Enhancement of blood supply	Diabetic retinopathy
Kim et al., 2015; Park et al., 2017 [100,102]	Protection against pathologic neovascularization	Diabetic retinopathy
Parisi et al., 2018 [103]	Anti-apoptosis and elevation of retinal dopamine level	Diabetic retinopathy

**Table 3:** Role of choline and choline metabolite in visual pathway.

References	Mode of Action or Mechanism	Organ
Parisi et al., 2018; Gandolfi et al., 2020 [103,117]	Restoration of mitochondrial functions and anti-apoptosis	Retinal ganglion cell and optic nerve
Parisi et al., 2019 [121]	Anti-degeneration	Optic nerve
Zhao et al., 2011 [124]	Cortical processing	Brain

## Data Availability

Not applicable.

## Abbreviations

ACh	Acetylcholine
AChE	Acetylcholinesterase
LDL	Low-density lipoprotein
